# Morphological adjustment in free-living *Steinernema feltiae* infective juveniles to increasing concentration of Nemafric-BL phytonematicide

**DOI:** 10.1371/journal.pone.0227448

**Published:** 2020-01-03

**Authors:** Phatu W. Mashela, Ebrahim Shokoohi, Kgabo M. Pofu

**Affiliations:** 1 University of Limpopo, Green Biotechnologies Research Centre of Excellence, Sovenga, Republic of South Africa; 2 Agricultural Research Council-VOP, Pretoria, Republic of South Africa; University of California Riverside, UNITED STATES

## Abstract

Third-stage larvae (L3) of *Steinernema feltiae* exist as free-living infective juveniles (IJ), with suspended development activities. In contrast, parasitic stages (L1, L2, L4, adult) have mutualistic relations with *Xenorhabdus* species bacteria, along with unique morphological changes and development inside the cadaver of host insects and/or plant-parasitic nematodes. Commercial IJ strains are tolerant to cucurbitacin-containing phytonematicides, but we have scant information on how morphological adjustments in IJ are achieved. In this study, we investigated the nature of morphological adjustments in commercial *S*. *feltiae* IJ strains to Nemafric-BL phytonematicide, which contains cucurbitacin B as active ingredient. Post-72 h exposure to phytonematicide concentration, IJ specimens were fixed on mounting slides. Length (body, excretory pore to anterior end, pharynx, rectum, stoma, tail), diameter (head width, neck base, mid-body, anal body), cuticle thickness and De Man ratios were measured with a computer software programme attached to Omax light microscope. Morphometric data against increasing phytonematicide concentration exhibited either density-dependent quadratic, linear or neutral relations. Increase in body length at low phytonematicide concentration was accompanied by decrease in tail length and pharynx length during muscle contraction when IJ were still alive. After death at high phytonematicide concentration, the opposite morphometric effects ensued due to muscle relaxation. The observed changes in morphometric structures were explained on the basis of morphological adjustments that modulated volumes of pseudocoelom cavity in IJ. The modulation is intended to maintain hydrostatic pressure within permissible upper limits in order to avoid structural damage to internal organs embedded in the pseudocoelom fluids.

## Introduction

Infective juveniles (IJ) in the Steinernematidae family are commercially used as potent entomopathogenic nematodes (EPN) in context of integrated pest management programmes, which are increasingly favoured in sustainable agro-cropping systems. EPN from Steinernematidae and Heterorhabditidae families have mutualistic relations with pathogenic bacteria in the genera *Xenorhabdus* and *Photorhabdus*, respectively, which confer host-specificity to insect and nematode stages in soil and aboveground [1–7]. In EPN the larvae (L1, L2, L3 and L4) and the adult occur as parasitic inside the host [8, 9]. However, when food resources begin to limit reproduction and development, the infective juveniles (IJ) exit the cadaver as L3 stage, with suspended bioactivities that confer this free-living stage the ability to withstand unfavourable conditions [8, 9, 10]. Post-infection of the living host, IJ undergo morphological changes, technically referred to as activation, thereby resuming feeding and development [9]. One major requirement in registration of agricultural biopesticides is that the materials should be compatible with EPN, along with effective microorganisms such as nitrogen-fixing bacteria.

Recent studies in our laboratory showed that the free-living commercial IJ strains of *Steinernema feltiae* (Filipjev 1934), were tolerant to Nemarioc-AL and Nemafric-BL phytonematicides within concentration ranges used for managing plant-parasitic nematode population densities [11, 12]. The two phytonematicides, with potent active ingredients cucurbitacin A (C_32_H_46_O_8_) and cucurbitacin B (C_32_H_46_O_9_), respectively, consistently suppress population densities of root-knot (*Meloidogyne* species) nematode and the citrus nematode, *Tylenchulus semipenetrans* Cobb 1913 [13]. Elsewhere, it was shown that *S*. *feltiae* IJ were also tolerant to neem (*Azadirachta indica* A. Juss 1830) products and the biofungicides *Streptomyces griseoviridis* and *Trichoderma harzianum* Rifai 1969 [14], along with synthetic chemical pesticides that include acetylcholine-inhibiting chlorpyrifos and primicarb nematicides [15, 16]. In bioactivity tests, within 0 and 9% phytonematicide concentration, IJ survival curves remained above mortality curves, with downward- and upward-facing slopes, respectively [11, 12]. The latter suggested that survival and mortality curves could eventually intersect as phytonematicide concentration continued to increase. In contrast, *Meloidogyne* species J2 against increasing concentration of cucurbitacin-containing phytonematicides consistently exhibited negative quadratic relations, with evidence suggesting that J2 were highly sensitive to the products [13, 17].

*Steinernema feltiae* IJ are commercially available in dauer-like stage for application in aqueous suspensions under favourable temperature conditions to promote active search for hosts [8, 9]. During host searching, *S*. *feltiae* IJ use an intermediate foraging strategy, which comprises a combination of ambush and cruiser type [18], that could last for at least a month [18], thereby having the potential to expose themselves to cucurbitacins in soil solution. Post-entry into the host, IJ undergo activation process which entails recovery from dauer-like, comprising fundamental changes that encompass gene expression for promoting transition from a free-living form to active parasitic larval stages [19]. During activation process, parasitic larval stages release *Xenorhabdus* species from their intestines into the hemocoel of hosts, with the bacteria multiplying in hemolymph of hosts, resulting in hosts dying within 24-48 h [7]. Thereafter, the developing stages continue to feed on bacterial cells and host tissues, with adults reproducing [9]. Depending on the availability of food resources, multiple life cycles occur within the cadaver, with thousands IJ eventually released out of the cadaver into soil solution to search for suitable hosts [9]. In soil environments where plant-parasitic nematodes are managed using cucurbitacin-containing phytonematicides in IPM programmes, the potential for exposing *S*. *feltiae* IJ to cucurbitacins is high. However, due to its tolerance to cucurbitacin-containing phytonematicides [11, 12] and other products with potent nematicidal properties [14–16, 20], it is probable that *S*. *feltiae* IJ could pick up chemical cues from such products and then reacted appropriately. *Steinernema feltiae* IJ could, therefore, serve as an ideal model for investigating the mechanism whereby EPN survive the exposure to a wide range of cucurbitacin-containing phytonematicide concentrations. In the current study we used *S*. *feltiae* IJ to investigate morphological adjustments under increasing concentration of Nemafric-BL phytonematicide during short-term exposure period equivalent to its free-living time-frame in aqueous solutions.

## Materials and methods

### Materials and experimental design

Mature fruit of *Cucumis africanus* were harvested from a cultivated field at 92 days after transplanting seedlings. Fruit were washed using chlorine-free tapwater, cut into pieces and dried at 52°C for 72 h [21]. Dried fruit were ground in a Wiley Mill Model 4 to pass through a 1-mm-pore sieve. Approximately 40 g ground material was fermented in hermetically-sealed 20-L-plastic containers (4 × containers) using effective microorganisms (EM) at 30°C as explained previously [13]. The EM culture comprised yeast, photosynthetic bacteria, lactic acid bacteria, actinomycetes and fermenting fungi [22]. Post-fermentation at 14 days, 1000 ml sample at pH 3.7 was passed through a Whatmann 1442-125 Ashless Grade 42 Quantitative Filter Paper. Dilutions at 0, 2, 4, 8, 16, 32 and 64% phytonematicide were made in hermetically sealed 150 ml glass measuring cylinders prior to temporary storage in 150 ml opaque plastic containers. The 0% phytonematicide comprised distilled water that served as a negative control.

Commercial strains of *S*. *feltiae* IJ in dauer-like state (Koppert Biological Systems, Johannesburg) were stored at 4°C prior to use. IJ were exposed to seven treatment concentrations following the modified method that was previously prescribed for *S*. *feltiae* IJ [11, 12]. Briefly, approximately 5 000 IJ were exposed to different concentrations in 96 well plates, as 200 μL solution containing IJ in suspension. IJ within seven treatments were arranged in an incubator at 21 ± 2°C in completely randomised design, with five replications (n = 35).

### Morphometric measurements

**Fig 1:**
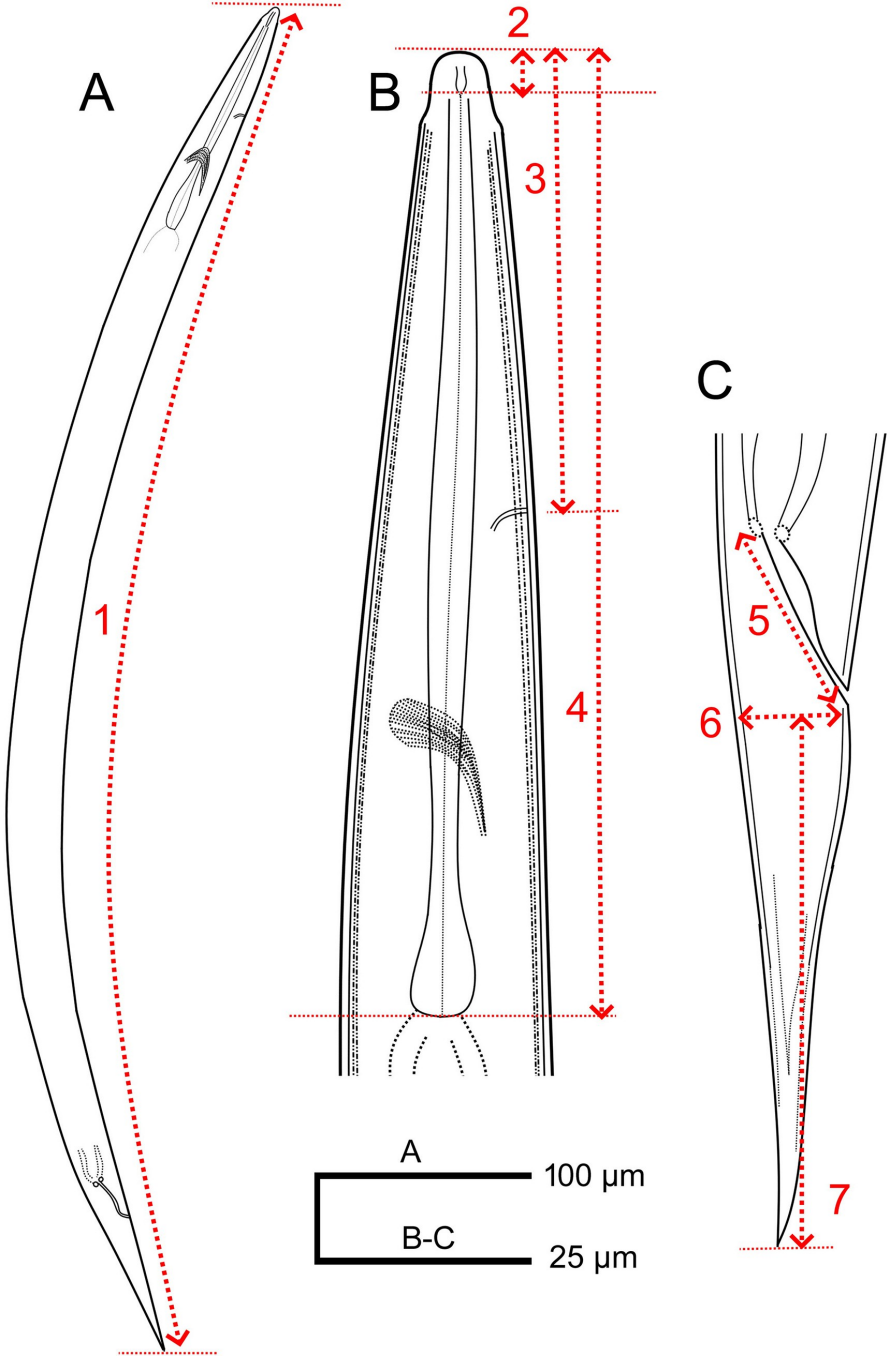
Morphological characters of *Steinernema feltiae* at zero concentration of Nemafric-BL phytonematicide. 1: Body length; 2: Stoma; 3: Excretory pore to anterior end; 4: Pharynx; 5: Rectum; 6: Anal body diameter; 7: Tail (All measurements were in μm).

At 72 h post-exposure, 50 μL solution with IJ suspensions per treatment was placed in a clean 96-well plates. Specimens were fixed on mounting slides [23] and morphometric data collected using Omax light microscope equipped with a measuring software programme. Morphometric data included length (body, excretory pore to anterior end, pharynx, rectum, stoma, tail), diameter (head width, neck base, mid-body, anal body) and cuticle thickness (Fig 1). Additionally, the measuring computer software generated De Man ratios [23], namely, *a*, *b*, *c*, *c'*, where ratio *a* = body length/mid-body diameter, ratio *b* = body length/pharynx length, ratio *c* = body length/tail length and ratio c' = tail length/to anal body diameter [Fig 1]. The experiment was validated in time within two weeks after the original.

### Data analysis

Prior to analysis of data, treatments (0, 2, 4, 8, 16, 32 and 64% phytonematicide) were expressed as exponentials [2^0^, 2^1^, 2^2^, 2^3^, 2^4^, 2^5^ and 2^6^ (%)] and transformed using log_2_2^x^ = x (log_2_2) = (x × 1) = x [24]. Almost always, without transformation, the generated quadratic curves were skewed to the right [24]. Data were then subjected to analysis of variance using Statistix computer software, along with means and the standard error of the means (SEM). Morphometric means for each variable (S1 File) against phytonematicide concentration were subjected to lines of the best fit using Microsoft Excel 2016. Findings were grouped and explained on the basis of their dose-response growth patterns [13]. Unless otherwise stated, treatment effects were discussed at the probability level of 5%.

## Results

Interactions of two-week time-frame between the original and validated experiments were not significant (p > 0.05). Data were therefore pooled (n = 70) and re-analysed as explained in the initial experiment.

### Positive quadratic responses

**Fig 2:**
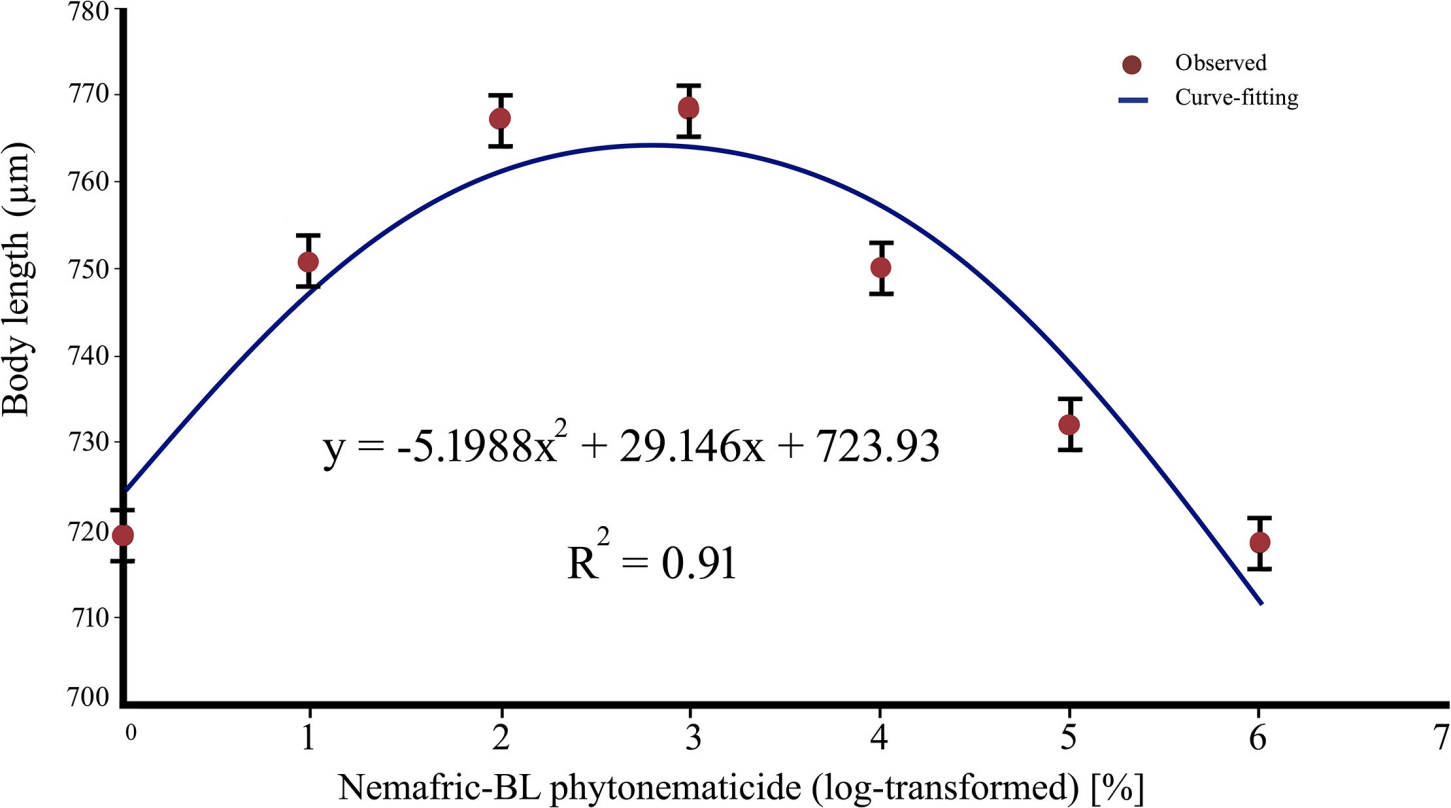
Positive quadratic curve for *Steinernema feltiae* body length against Nemafric-BL phytonematicide, with log-transformed (untransformed) extremum at 2.80% (5.60%).

**Fig 3:**
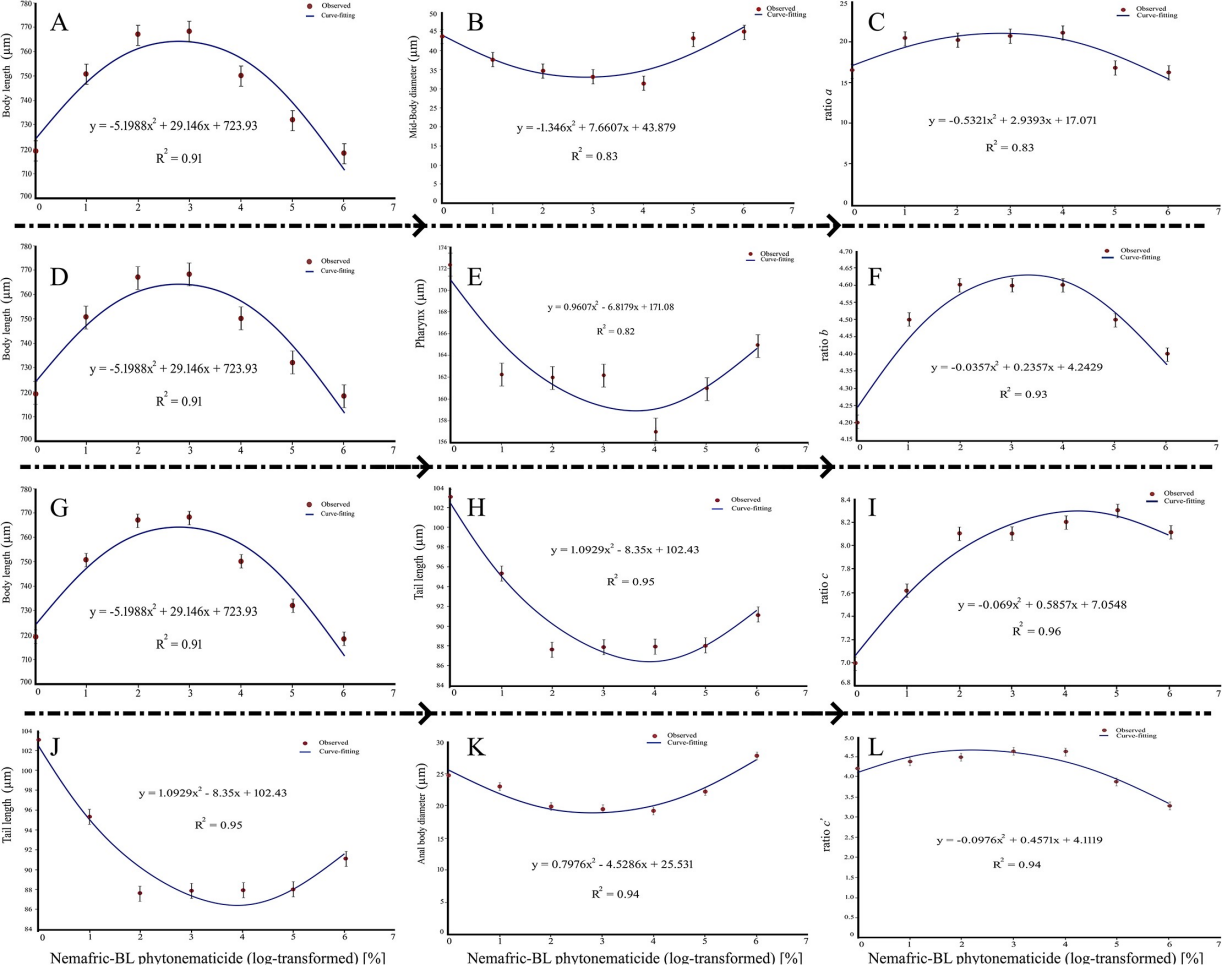
Positive quadratic relations in *Steinernema feltiae* De Man ratios versus Nemafric-BL phytonematicide: Ratio a (C = A/B) as proportion of body length (A) to mid-body diameter (B), ratio b (E = D/E) as proportion of body length (D) to pharynx length (E), ratio c (I = G/H) as proportion of body length (G) to tail length (H) and ratio c' (L = J/K) as proportion of tail length (J) to anal body diameter (K).

The body length of *S*. *feltiae* IJ against increasing concentration of Nemafric-BL phytonematicide exhibited positive quadratic relations, with the model being explained by 91% coefficients of determination (CsOD) (Fig 2). Optimum concentration for body length, derived using x = –b_1_/2b_2_ relation [25], was at 5.60% untransformed phytonematicide, beyond which the variable started to decrease. Similarly, De Man ratios a, b, c and c' against increasing phytonematicide concentration each exhibited positive quadratic relations, with CsOD explaining the models by 83% (Fig 3A), 93% (Fig 3B), 96% (Fig 3C) and 94% (Fig 3D), respectively. The respective optimum extrema for the ratios ranged from 4.68 to 8.49% (Table 1), with average value of 6.32% phytonematicide, which was equivalent to that of the optimum body length.

**Table 1:** Extrema for Nemafric-BL phytonematicide concentration to generate optimum/minimum morphometrics for organs in *Steinernema feltiae* infective juveniles.

Optimum		Minimum			
De Man ratio	x (%)^z^	Organ (μm)	x (%)	Organ (μm)	x (%)
A	2.76 (5.52)	Neck base diameter	2.59 (5.18)	Tail length	3.82 (7.64)
B	3.30 (6.60)	Mid-body diameter	2.85 (5.69)	Head width	3.30 (6.60)
C	4.24 (8.49)	Stoma length	2.68 (5.36)	Cuticle thickness	2.63 (5.26)
c'	2.34 (4.68)	Anal body diameter	2.84 (5.68)	Pharynx	3.55 (7.10)
$\bar{x}$ = 3.16 (6.32)			$\bar{x}$ = 3.03 (6.06)		

^z^Extrema for log-transformed data (outside bracket) and untransformed data (inside bracket) data.

### Negative quadratic responses

**Fig 4:**
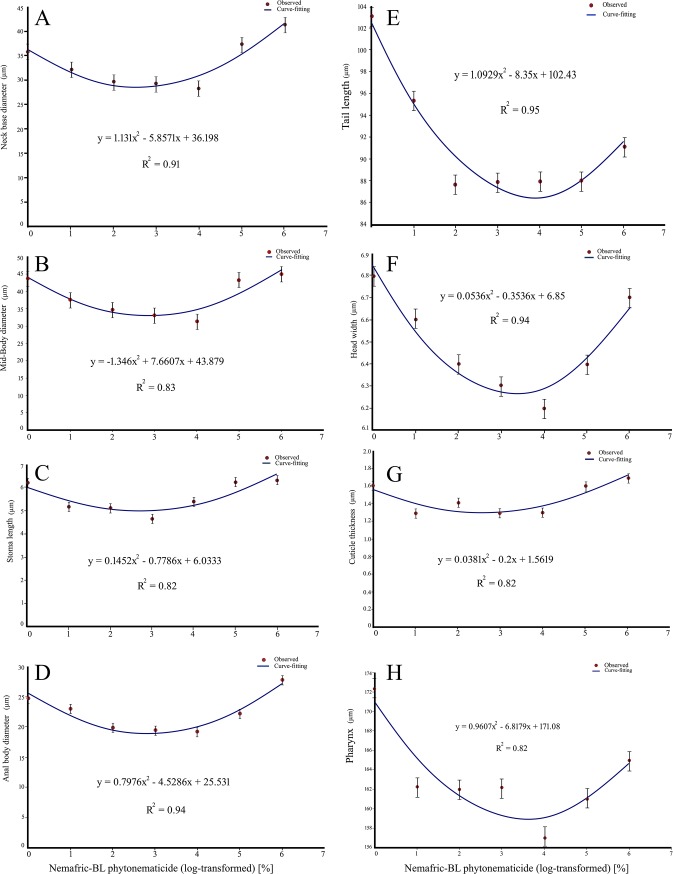
Negative quadratic relations in *Steinernema feltiae* selected morphometric against Nemafric-BL phytonematicide: Neck base diameter (A), mid-body diameter (B), stoma length (C), anal body diameter (D), tail length (E), head width (F), cuticle thickness (G) and pharynx length (H).

Neck base diameter, mid-body diameter, stoma length and anal body diameter versus Nemafric-BL phytonematicide exhibited negative quadratic relations, with models explained by 91% (Fig 4A), 83% (Fig 4B), 82% (Fig 4C) and 94% (Fig 4D) CsOD, respectively. Similarly, tail length, head width, tail length, cuticle thickness and pharynx length versus Nemafric-BL phytonematicide each exhibited negative quadratic relations, with the models being explained by 95% (Fig 4E), 94% (Fig 4F), 82% (Fig 4G) and 82% CsOD (Fig 4H), respectively. Generally, as concentration increased, organs gradually declined to the minimum and after the nematode has died, the organs started to increase again. Morphometric variables that had negative quadratic relations with Nemafric-BL phytonematicide attained their minimum values from 5.18 to 7.64% phytonematicide (Table 1), with average value of 6.06% phytonematicide.

### Negative linear and neutral responses

**Fig 5:**
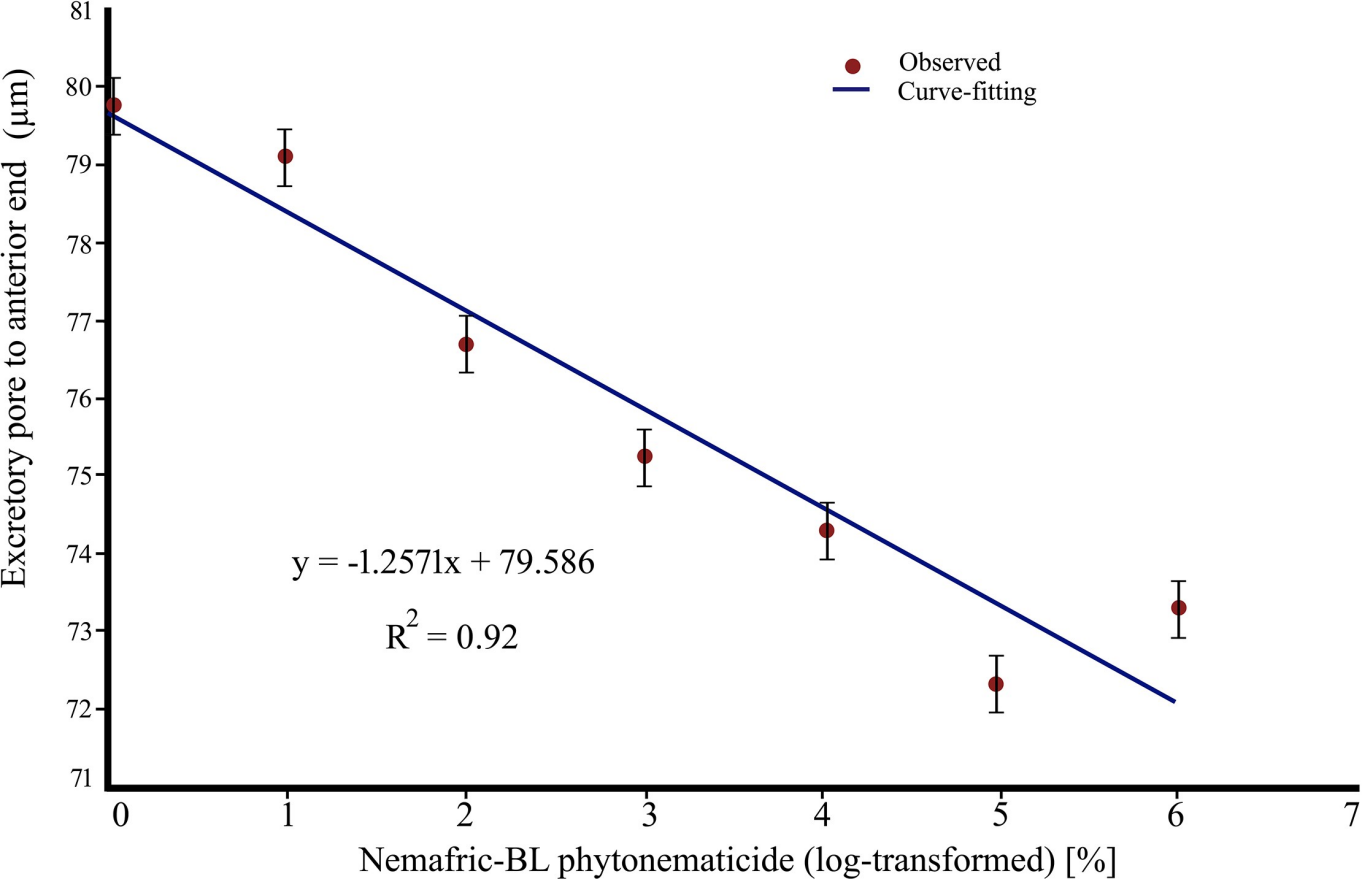
Negative linear relation in *Steinernema feltiae* excretory pore to anterior end length versus Nemafric-BL phytonematicide.

The excretory pore to anterior end length versus Nemafric-BL phytonematicide exhibited negative linear relations, with the model explained by 92% coefficient of determination (Fig 5). The rectum versus the phytonematicide concentration did not have significant relationship (r = 0.38). Neutral responses are common in allelochemical-exposed entities [13, 24].

## Discussion

Observations in the study demonstrated various adjustments in the morphology of *S*. *feltiae* free-living IJ gradually increased at low concentrations of test phytonematicide. Approximately 6% phytonematicide was the optimisation concentration, beyond which adjustments entered the saturation phase, characterised by static adjustments, where further increase in concentration within the phase had no significant effects on adjustments. However, as phytonematicide concentration increased beyond the phase, the affected adjustments gradually declined. These density-dependent response adjustments are common in most entities that have been exposed to increasing concentration of allelochemicals [26]. The three phases, namely, stimulation, neutral and inhibition phases [26], were pronounced for body length and De Man ratios of *S*. *feltiae* IJ in the current study. However, in plant-parasitic nematodes that were exposed to similar geometric series of increasing phytonematicide concentration, the body length entered the inhibition phase with immediate effect, due to paralysis and death [13]. In the current study we argue that the morphometric adjustment in body length of *S*. *feltiae* IJ at low phytonematicide concentration was intended to regulate the volume of pseudocoelomic cavity in order to maintain the hydrostatic pressure in equilibrium [27]. Such adjustments can be viewed as being a modification of the pseudocoelomic cavity size to ensure that the hydrostatic pressure does not overstep the permissible upper limit [27], where damage to internal organs would be irreversible.

Generally, hydrostatic pressure originating from body fluids in pseudocoelomic cavity pushes against the cuticle, which in turn pushes the body fluids with the same force (*Newton’s third law*) being exerted against internal organs. The hydrostatic pressure gives the nematode its cylindrical body shape [27] and has been quantified during active and passive bioactivities of *Ascaris lumbricoides* (Linnaeus 1758) [27], which has L3 as IJ [10]. Results in the current study agreed with those in *A*. *lumbricoides* as a zoo-parasitic nematode, where adjustments in body length were ascribed to contraction of longitudinal somatic muscles, with subsequent quadratic reduction in cuticle thickness, pharynx length and tail length [27].

**Fig 6:**
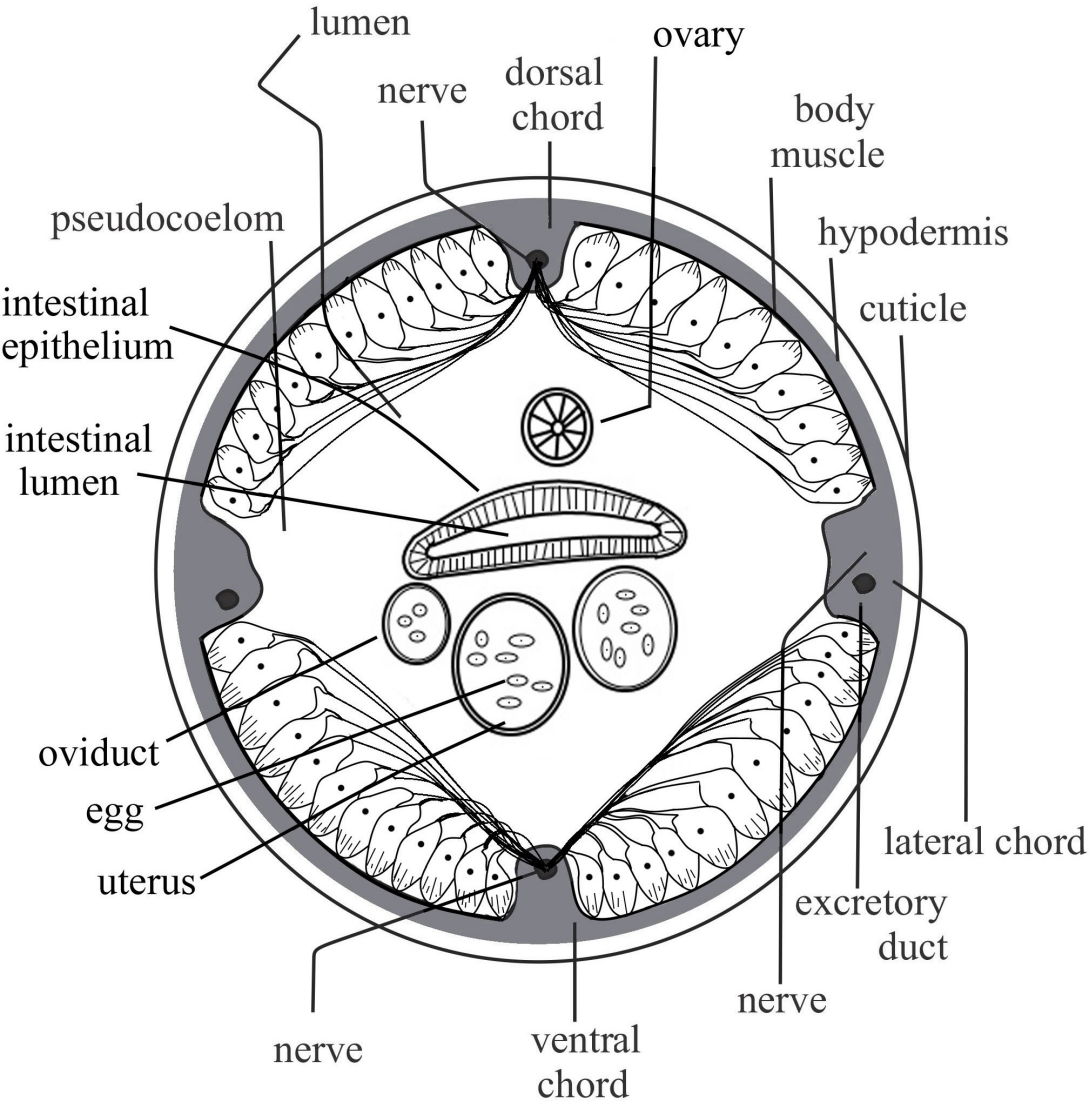
Cross-section of a nematode showing cuticle, muscles and internal organs in pseudocoelom cavity (Improved from Shokoohi, 2019).

Longitudinal somatic muscles in nematodes occur as separate pear-like structures [28], as opposed to long striated muscles in high animals. The nematode muscles have three distinct parts, namely, (a) spindle with contractile muscle filament lattice (*basal part of pear*), (b) non-contractile muscle cell body (*body of pear*) with sarcoplasmic organelles and nucleus and (c) arms (*fruit-stalk end of pear*) extending from the latero-dorsal longitudinal somatic muscles to the nerve of the dorsal chord, whereas those from arms of the latero-ventral muscles extend to the nerve of the ventral chord (Fig 6) [29]. The pear-shaped muscles in longitudinal rows are arranged on the latero-dorsal and the latero-ventral spaces, with the non-contractile regions submerged in body fluid of the pseudocoelomic cavity (Fig 6). The muscles occur in rows of either 2-5 (*meromyarian*) or more than five (*polymyarian*) [28]. Typically, spindles (contracting parts) are embedded on the hypodermal layer of cuticle, thereby contracting against the elastic cuticle that is made rigid by the hydrostatic pressure [30]. Contraction of spindle regions increases the volume of non-contractile somatic cell bodies in the pseudocoelom cavity, thereby decreasing its volume and at the same time increasing the hydrostatic pressure. Unless there was some form of body adjustment that increases the pseudocoelom cavity volume, the hydrostatic pressure would shoot above the permissible upper limit, which in *A*. *lumbricoides* was equivalent to 225 mm Hg (= 29.99753 kPa) [27].

Due to the symmetric nature of the latero-dorsal and latero-ventral longitudinal somatic muscles, when the latero-dorsal muscles contract in a given region (*increase in muscle volume*), equivalent rows of the latero-ventral somatic muscles relax (*decrease in muscle volume*), results in the nematode body being bent towards the ventral side, *vice versa*. The simultaneous contraction-relaxation of opposite longitudinal somatic muscles modulates potential excessive hydrostatic pressure in pseudocoelom cavity of nematode body (Fig 6). The simultaneous relaxation and contraction of longitudinal somatic muscles in opposite symmetries, results in nematode bodies bending towards the symmetry with relaxed muscles. In *S*. *feltiae* IJ, nictation in during host searching [19] is the result of simultaneous relaxation and contraction of longitudinal somatic muscles towards the anterior end of the nematode. In contrast, simultaneous contraction of latero-dorsal and latero-ventral longitudinal somatic muscles in a particular region of the nematode body, results in that part of the body being straightened [28]. Similarly, when simultaneous contraction of longitudinal somatic muscles occurs in both symmetries along the entire nematode body, elongation that increases body length occurs. The elongated body is intended to modulate effects of increased hydrostatic pressure caused by increased volumes of non-contractile portions of muscles due to contraction of the spindles. Increase in body length avoids damage to complex internal organs within the pseudocoelom space such as excretory, digestive, nervous and reproductive systems [28].

In our study, the body length elongated in the stimulation phase, which is supported by decreases in diameters of various body parts. Increases in body length and decreases in certain organs, modulate effects of hydrostatic pressure that emanates from simultaneous contraction longitudinal somatic muscle cells from opposite symmetrical spaces. The contraction invariably results in increased volumes of non-contractile muscles, thereby increasing hydrostatic pressure in pseudocoelom spaces as empirically-demonstrated in *A*. *lumbricoides* [27]. Increases in body length during stimulation phase of the phytonematicide as observed in the current study, is part of survival strategy used by *S*. *feltiae* IJ. The stimulation phase is followed by the neutral phase, where increasing phytonematicide concentration has neutral effects on body elongation. Beyond the neutral phase, as phytonematicide concentration increases further, the body length enters the inhibition phase, denoting death and simultaneous relaxation of longitudinal somatic muscles on both symmetrical spaces, with body length decreasing as shown by increasing diameters of various body organs in the current study.

Ratio *a*, for instance, is a proportion of body length to mid-body diameter [23]. The two variables responded differently to phytonematicide concentration, with body length against phytonematicide exhibiting positive quadratic relation, whereas mid-body diameter against phytonematicide exhibited negative quadratic relation. Consequently, at low concentration, as body length increased, the mid-body diameter decreased, resulting in increasing ratio *a*, whereas at high concentration, as body length decreased, mid-body diameter increased, thereby reducing the ratio. Similar trends were observed in ratios *b* and *c*. In contrast, for ratio *c'*, as both tail length and body diameter decreased with increasing phytonematicide concentration, the ratio increased and eventually decreased as the components increased. The changes in the ratios is further evidence of adjustments that affected the entire nematode body.

The negative quadratic response for diameters as concentrations increased was congruent with the advanced hypothesis on nematode body length as depicted through the three density-dependent growth phases. At low concentration as body length increased, body diameters decreased, whereas at high concentration as body length decreased after death, diameters increased. After exposure to approximately 6% Nemafric-BL phytonematicide, *S*. *feltiae* IJ were irreversibly damaged, whereas at low phytonematicide concentration used to manage plant-parasitic nematodes, morphometric adjustments occurred. Additionally, quadratic decreases in head width and stoma length of *S*. *feltiae* IJ were congruent with increase in body length at low phytonematicide concentration, with increase in head width being observed as body length decreased at high concentration. Also, quadratic decreases in pharynx length and tail length as the body length increased, confirming the presence of a different set of specialised muscles in these organs [28], which do not operate in consonant with longitudinal somatic muscles [27]. In all cases, the quadratic decreases in the two organs as the body length increases provide additional pseudocoelomic volume for body fluids, with consequent effects being to modulate hydrostatic pressure in order to avoid detrimental effects on internal organs.

The cuticle comprises a set of six layers [28], with the outermost thin layer of glycoproteins and lipids, with annuli and furrows [30]. Cucurbitacins are lipophilic and therefore, the cuticle is predisposed to cucurbitacin attack and damage [31]. Generally, cucurbitacins interfere with the integrity of cell membranes, which are further away in the innermost basal layer. The structure of the cuticle, with collagen struts serving as pillars that separate the outermost and innermost layers, along with the fluid-filled space [27, 28]. All confer the body the capability to stretch longitudinally by modifying angles of the fibrous layers that enhance the nematode body to have the hydrostatic skeleton [28]. In organs with morphometrics that exhibited negative quadratic relations, after the extremum, which depicted the phytonematicide concentration at which the nematode was dead, morphometrics exhibited slight “increasing trends”. Since cucurbitacins interfere with the integrity of the cuticle [32], resulting in loss of semi-permeability of membranes. Due to high solutes of fluids in the pseudocoelom cavity, water tends to move into the nematode bodies, thereby giving pseudo-increments in morphometrics of certain organs. In *S*. *feltiae* IJ the excretory pore is located on the ventral side anterior to the nerve ring [1]. The distance, excretory pore to the anterior end, against increasing phytonematicide concentration exhibited negative linear relations. Basically, this observation confirmed the observed decreases in pharynx length and stoma length as phytonematicide concentration increased. Interestingly, as *S*. *feltiae* IJ died at high phytonematicide concentration, the damage became irreversible, as observed in other nematodes where damage to the excretory pore was irreversible [27]. The rectum (r = 0.38) against increasing phytonematicide concentration did not have significant relationship. In context of dose-response growth patterns induced by allelochemicals [26], neutral responses suggest that the variable was saturated with cucurbitacin B at sampling [13]. Such responses are common when entities are exposed to increasing concentration of phytonematicides [13].

## Conclusion

The increase in body length and decrease in head diameter, pharynx length, body width diameter, tail length, excretory pore to the anterior, anal body diameter and stoma length at low concentration of Nemafric-BL phytonematicide, all supported the view of body adjustments as a tolerant strategy. Generally, within the concentration range that Nemafric-BL phytonematicide is used to manage population densities of plant-parasitic nematodes, *S*. *feltiae* IJ have the capability to adjust various morphological structures in order to avoid fatal damage to internal organs induced by increasing hydrostatic pressure of body fluids in pseudocoelom cavity.

## Supporting information

S1 File Morphometric data file of *Steinernema feltiae* versus increasing concentration of Nemafric-BL phytonematicide. (XLSX)
